# Gadolinium ethoxybenzyl diethylenetriamine pentaacetic acid-enhanced magnetic resonance imaging: A potential utility for the evaluation of regional liver function impairment following transcatheter arterial chemoembolization

**DOI:** 10.3892/ol.2014.2826

**Published:** 2014-12-23

**Authors:** YU-DONG XIAO, RAMCHANDRA PAUDEL, HUAN LIU, BIN ZHANG, CONG MA, SHUN-KE ZHOU

**Affiliations:** Department of Radiology, The Second Xiangya Hospital of Central South University, Changsha, Hunan 410011, P.R. China

**Keywords:** magnetic resonance imaging, regional liver function, gadolinium ethoxybenzyl diethylenetriamine pentaacetic acid, transcatheter arterial chemoembolization

## Abstract

The present study aimed to evaluate regional liver function impairment following transcatheter arterial chemoembolization (TACE), assessed by magnetic resonance imaging (MRI) enhanced by gadolinium ethoxybenzyl diethylenetriamine pentaacetic acid (Gd-EOB-DTPA). Additionally, this study evaluated the associations between signal intensity and various clinical factors. A prospective study was conducted between March 2012 and May 2013 with a total of 35 patients. Gd-EOB-DTPA-enhanced MRI was performed 3–5 days after TACE therapy. The signal to noise ratio (SNR) was subsequently calculated for healthy liver tissue regions and peritumoral regions, prior to and 20 min after Gd-EOB-DTPA administration. The correlation between clinical factors and relative SNR was assessed using Pearson’s correlation coefficient or Spearman’s rank correlation coefficient. Prior to Gd-EOB-DTPA administration, the SNR values showed no significant difference (t=1.341, P=0.191) in healthy liver tissue regions (50.53±15.99; range, 11.25–83.46) compared with peritumoral regions (49.81±15.85; range, 12.34–81.53). On measuring at 20 min following Gd-EOB-DTPA administration, the SNR in healthy liver tissue regions (82.55±33.33; range, 31.45–153.02) was significantly higher (t=3.732, P<0.001) compared with that in peritumoral regions (75.77±27.41; range, 31.42–144.49). The relative SNR in peritumoral regions correlated only with the quantity of iodized oil used during TACE therapy (r=0.528, P=0.003); the age, gender, diameter and blood supply of the tumor, or Child-Pugh class of the patient did not correlate with relative SNR. Gd-EOB-DTPA-enhanced MRI may be an effective way to evaluate regional liver function impairment following TACE therapy.

## Introduction

Hepatocellular carcinoma (HCC) is one of the most common types of liver cancer, with ~748,300 new liver cancer cases and 695,900 liver cancer-related mortalities occurring worldwide (1). The overall five-year survival rate of HCC is 3% in the USA (2). A population-based study conducted by Guiu *et al* reported that the one-year survival rate of HCC had increased to 32.8% and the five-year survival rate of HCC had risen to 10.0% over the past four decades (3). Surgical resection is the first option for HCC patients who meet the Milan Criteria (4): (i) one lesion <5 cm; (ii) ≤3 lesions <3 cm; (iii) no extrahepatic manifestations and (iv) no vascular invasion. However, it is not feasible when patients present at an advanced stage of the disease (5). Furthermore, conservative treatments, including chemotherapy, radiotherapy and biotherapy may not achieve satisfactory curative results (6). Transcatheter arterial chemoembolization (TACE) is the primary treatment option for patients with unresectable HCC (7). Approximately 16–55% patients can benefit from TACE therapy and achieve a low rate of tumor regression. Llovet *et al* reported that TACE resulted in objective responses that were sustained for ≥6 months in 35% of cases, and was associated with a lower rate of portal-vein invasion compared with conservative treatment (8). Following TACE therapy, HCC tumor cells undergo ischemia and necrosis. Healthy liver tissue is inevitably damaged during the procedure, which may adversely affect the postoperative recovery of the patient (9). Magnetic resonance imaging (MRI) in combination with liver-specific contrast agents facilitates the detection of focal liver disease, and has been demonstrated to be superior to computed tomography (CT) for this purpose (10). Gadolinium ethoxybenzyl diethylenetriamine pentaacetic acid (Gd-EOB-DTPA; Primovist), is a liver-specific, paramagnetic contrast agent developed by Bayer-Schering Pharma (Berlin, Germany) with combined perfusion and hepatocyte-selective properties. A number of studies have demonstrated the reliability of Gd-EOB-DTPA as a non-invasive tool for estimating overall and regional liver cell function and viability, through measuring the cytomembrane transporter function (such organic anion transporting polypeptide 1 and MRP) (11–13). The present study aimed to investigate the potential utility of Gd-EOB-DTPA-enhanced MRI in the evaluation of regional liver function damage in peritumoral regions following TACE therapy.

## Materials and methods

### Ethics statement

Written, informed consent was obtained from all patients. The study was conducted in accordance with the Declaration of Helsinki and approved by the ethics committee of The Second XiangYa Hospital of Central South University (Changsha, China).

### Patients

A total of 35 HCC patients who underwent Gd-EOB-DTPA-enhanced MRI of the liver were enrolled in this prospective study between March 2012 and May 2013. Of the 35 patients, four were excluded from the study due to poor postoperative clinical status and two were excluded due to poor breath-holding during MRI examination. The final study population comprised 29 patients [18 males and 11 females; mean age ± standard deviation (SD), 49.86±11.05 years; age range, 28–76 years].

The diagnosis of HCC was determined on the basis of the following criteria: Typical lesions observed on at least one imaging modality (CT, MRI or ultrasound) with an elevated serum α-fetoprotein level (>400 ng/ml; n=23) or liver biopsy with pathological confirmation of HCC (n=12).

Inclusion criteria were as follows: Patients who could understand the study documents (which contained the study design, content, background, methods and possible treatment outcomes), had a single lesion with tumors <10 cm in size; were categorized as Child-Pugh class A or B prior to surgery and were receiving TACE therapy for the first time. Exclusion criteria were as follows: Severe motion artifacts due to poor breath-holding, profound liver cirrhosis, multiple lesions, tumors >10 cm in size, obstruction of main or first branch of portal vein, difficulty in locating the feeding artery during the procedure or hepatic artery to portal vein shunting. Exclusion criteria for the use of Gd-EOB-DTPA were as follows: Allergy to Primovist, severe cardiovascular disease or kidney insufficiency (glomerular filtration rate <60 ml/min/1.73 m^2^).

### TACE protocol

TACE was performed via the femoral artery under GELCE 3100 (GE Healthcare, Fairfield, USA) bidirectional digital subtraction angiography. Using the Seldinger technique (14), a catheter sheathe was inserted into the femoral artery with the aid of a guide wire. The Yashiro, RH catheter or Microcatheter (Terumo, Tokyo, Japan) was sent to the artery feeding the tumor. The Yashiro and RH catheters were the first option for TACE therapy; when these two catheters were not effective, the microcatheter was used, as it is smaller than the Yashiro and RH catheters. However, the microcatheter was not regularly used, owing to its high cost. Iodized oil (5–30 ml; mean, 16.14±7.04 ml; Guerbet, Paris, France) mixed with chemotherapeutics (pirarubicin, 10–40 mg; 5-fluorouracil, 250–1,000 mg; cisplatin, 40–80 mg) was injected into the feeding artery of the tumor.

### MRI protocol

MRI was performed 3–5 days after TACE therapy using a 3T superconducting MRI system (Philips, Amsterdam, Netherlands) with a phased array body coil (SENSE XL Torso; Philips) and the following imaging parameters: 7 mm section thickness and 3 mm intersection gap. Three-dimensional T1-weighted turbo field echo sequence with spectral presaturation inversion recovery fat suppression [repetition time (TR), 3.0 ms; echo time (TE), 1.35 ms; field of view, 350×320 mm; matrix, 124×100; flip angle, 10°] was utilized pre-contrast and post-contrast (15 s, 90 s, 3 min and 20 min after injection of Gd-EOB-DTPA). Respiratory-triggered T2-weighted fast spin echo sequence with short TI inversion recovery fat suppression (TR, 1,113 ms; TE, 70 ms; field of view, 350×320 mm; matrix, 268×200; flip angle, 90°) was used prior to injection of the contrast agent. The contrast agent was used at a dose of 0.025 mmol/kg body weight and at an injection rate of 2 ml/s by 20 ml saline flush using an intravenous line (via the cubital vein).

### Imaging analysis

**Figure 1:**
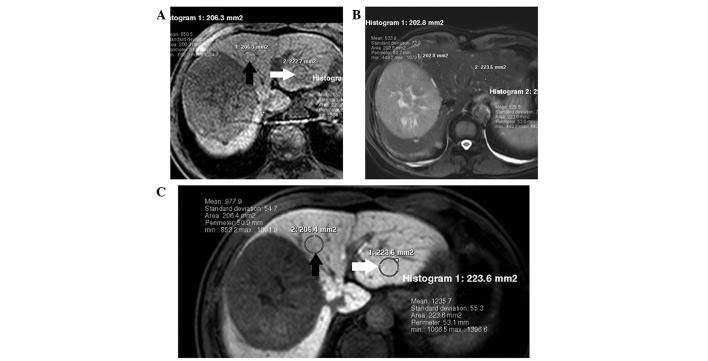
(A and C) Signal to noise ratio was calculated for peritumoral regions (black arrows) and healthy tissue regions (white arrows) prior to (A) and 20 min after (C) gadolinium ethoxybenzyl diethylenetriamine pentaacetic acid administration. (B) T2-weighted magnetic resonance imaging revealed a lesion in the peritumoral region exhibiting no significantly high signal intensity.

In the evaluation of hepatocytic uptake of Gd-EOB-DTPA, signal intensity (SI) of the region of interest (ROI) of the liver was measured by one radiologist with 20 years experience of abdominal imaging, who was blinded to the clinical data and selection of ROIs. The ROIs were selected to be as large as possible, avoiding large vessels and biliary ducts, and the identical location was used prior to and following Gd-EOB-DTPA administration. Each ROI was circular or oval. Signal to noise ratio (SNR) was calculated for peritumoral and healthy liver tissue regions (Fig. 1), by dividing the SI of the tissue by the standard deviation of the image noise (background noise outside of the patient’s body).

The relative SNR in the peritumoral regions was measured to assess the correlation between hepatocytic uptake of Gd-EOB-DTPA and potential clinical influencing factors (patients age, gender, diameter of the tumor, blood supply of the tumor, Child-Pugh class and quantity of Iodized oil used). Relative SNR = [(SNR_after_ - SNR_before_)/SNR_before_] × 100.

### Statistics

The data are presented as the mean ± SD (range) and were analyzed using SPSS 19.0 software (SPSS Inc., Chicago, IL, USA). The correlation between clinical factors and relative SNR was analyzed using Pearson’s correlation coefficient or Spearman’s rank correlation coefficient. Pearson’s correlation analysis was used to evaluate the association between the relative SNR of liver parenchyma and the age of the patient, the diameter of the tumor and the quantity of iodized oil used for TACE therapy. Spearman’s rank correlation was used to evaluate the association between the relative SNR of liver parenchyma and clinical parameters including gender, blood supply of the tumor and Child-Pugh class. The quantitative parameter of healthy liver tissue regions vs. peritumoral regions was calculated using a paired *t*-test. P<0.05 was considered to indicate a statistically significant difference.

## Results

### Assessment of clinical influencing factors

Of the 29 patients, 22 were categorized as Child-Pugh class A and seven as Child-Pugh class B. The quantity of iodized oil used for individual patients varied from 5–30 ml, with a mean quantity of 16.14±7.04 ml. A poor blood supply to the tumor was observed in nine patients, while a rich blood supply was observed in the remaining 20. The diameter of the tumors ranged from 3.1–9.9 cm, with a mean diameter of 6.94±2.15 cm. No correlation was observed between the blood supply and the diameter of the tumor (r=0.276, P=0.148). Detailed clinical information is listed in Table I.

### Gd-EOB-DTPA uptake in different liver tissue regions

**Figure 2:**
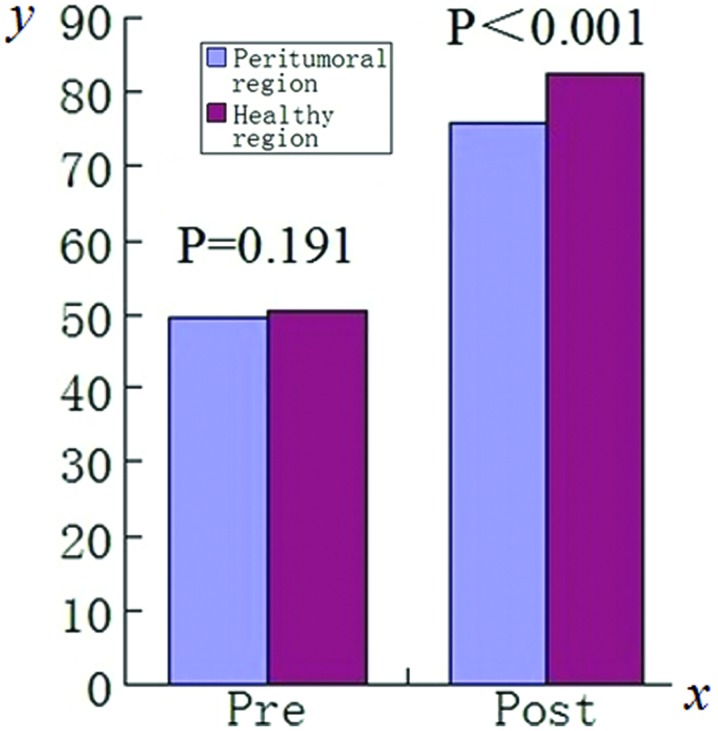
Prior to Gd-EOB-DTPA injection, no significant difference in SNR values was observed in healthy regions compared with peritumoral regions (P=0.191). 20 min after Gd-EOB-DTPA injection, the SNR in healthy regions was significantly higher compared with that of peritumoral regions (P<0.001). Gd-EOB-DTPA, gadolinium ethoxybenzyl diethylenetriamine pentaacetic acid; SNR, signal to noise ratio.

**Figure 3:**
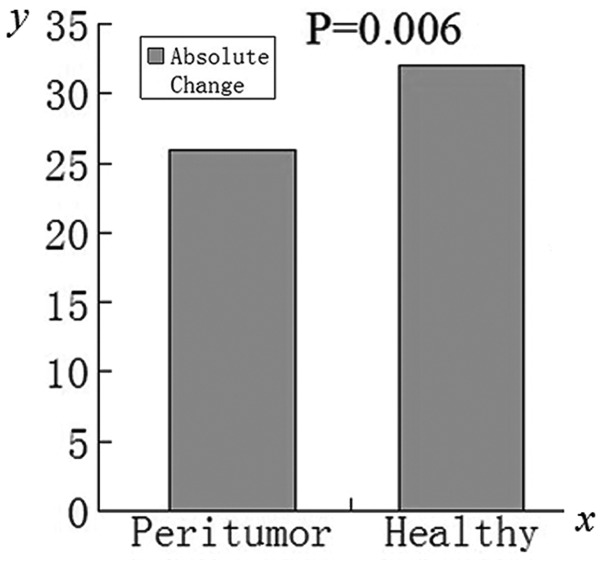
The absolute change in signal to noise ratio was observed to be significantly higher for healthy liver tissue regions compared with peritumoral regions (P=0.006).

Prior to Gd-EOB-DTPA administration, no significant difference was observed in the SNR values of healthy liver tissue regions (50.53±15.99; range, 11.25–83.46) compared with those of peritumoral regions(49.81±15.85; range, 12.34–81.53; *t*=1.341, P=0.191). When measured 20 min after the administration of Gd-EOB-DTPA, the SNR values of healthy liver tissue regions (82.55±33.33, range 31.45–153.02) were significantly higher compared with those of the peritumoral regions (75.77±27.41, range 31.42–144.49; *t*=3.732, P<0.001; Fig. 2). Further detail is shown in Table II. The SNR measured in healthy liver tissue regions was significantly increased at 20 min after the administration of Gd-EOB-DTPA compared with that prior to its administration (*t*=6.175, P<0.001). In peritumoral regions, the SNR also exhibited a significant increase when measured 20 min after Gd-EOB-DTPA administration (*t*=6.844, P<0.001) compared with that prior to administration. The absolute change in SNR for healthy liver tissue regions was significantly higher (*t*=3.005, P=0.006) compared with those of the peritumoral regions (Fig. 3).

### Association between relative SNR and its potential influencing factors

**Figure 4:**
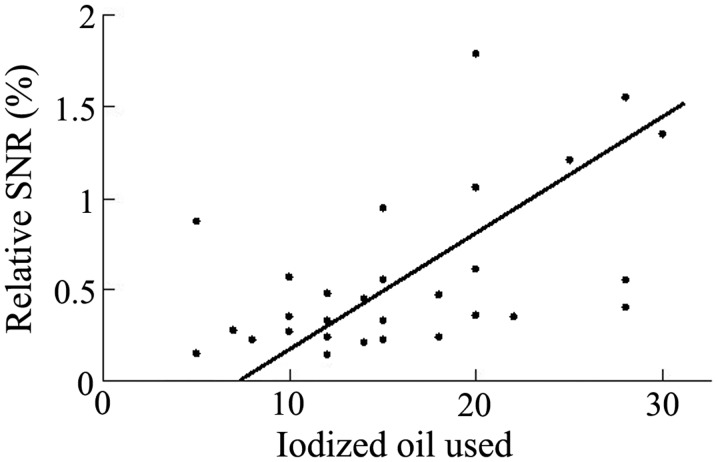
Scatter plot of the quantity (in ml) of iodized oil used for transcatheter arterial chemoembolization therapy and the relative SNR measured in peritumoral regions in the hepatobiliary phase images obtained 20 min after injection (r=0.528, P=0.003). SNR, signal to noise ratio.

The relative SNR did not correlate with the age (r=0.151, P=0.434), gender (r=−0.381, P=0.055) or Child-Pugh class (r=0.106, P=0.584) of the patient. Additionally, no correlation was observed between the SNR and the blood supply (r=0.241, P=0.209) or the diameter (r=0.226, P=0.238) of the tumor. Relative SNR, measured 20 min following Gd-EOB-DTPA administration, was observed to correlate only with the quantity of iodized oil used during TACE therapy (r=0.528, P=0.003; Fig. 4). Further detail is shown in Table III.

## Discussion

As TACE therapy may damage normal liver tissue, patients with pre-existing liver dysfunction commonly experience hepatic failure following TACE therapy (15). A previous study conducted by Chen *et al* suggested that the liver function after TACE therapy was significantly decreased compared with the preoperative status (16). Injuries caused by TACE therapy may affect the selection of the surgical procedure for patients subsequently requiring tumor resection (17). Reliable estimation of regional liver function in the preoperative and postoperative periods is crucial, particularly for patients at high risk of hepatic failure. Shimizu *et al* (18) evaluated regional liver function in a rat ischemia-reperfusion model by Gd-EOB-DTPA-enhanced MRI. Ischemic lobes were visualized as areas of high signal intensity in the hepatobiliary region. A retrospective study conducted by Yamada *et al* (19) suggested that liver function, corresponding to the plasma disappearance rate of indocyanine green, could be estimated by Gd-EOB-DTPA-enhanced MRI; this may improve the assessment of segmental liver function. The current study aimed to evaluate the impairment of regional liver function to provide guidance for the prevention and treatment of hepatic insufficiency in the selected patients, subsequent to TACE therapy.

Hepatocytic uptake of Gd-EOB-DTPA is hypothesized to be regulated by an active membrane transport system such as OATP, particularly OATP1B1 and OATP1B3, which require adenosine triphosphate (ATP) for their activity (20). These two transporters are also able to transport bilirubin (21). Bilirubin is the main pigment in human bile and thus, it may be used to indicate the quality of liver function. As these two transporters are also able to transport serum bilirubin, the uptake of Gd-EOB-DTPA by hepatocytes is associated with the serum levels of bilirubin. As the deposition of embolic agents can significantly reduce the blood supply of the hepatic artery, the peritumoral liver tissue is inevitably damaged during the TACE procedure by ischemia and hypoxia of hepatocytes (22,23). Such injury may cause a decrease in ATP production, leading to the functional limitation of transporters, which may be the reason for the reduction in uptake of Gd-EOB-DTPA (24). In addition, it has been reported that decreased expression of OATP1B1 and OATP1B3 transporters may lead to reduced Gd-EOB-DTPA uptake by hepatocytes in rat models (25).

Several studies have proposed that insufficient liver parenchymal enhancement in Gd-EOB-DTPA-enhanced imaging may indicate liver dysfunction (26–28). A cohort study conducted by Utsunomiya *et al* (29) reported that utilization of Gd-EOB-DTPA with MRI may provide a potential method for the estimation of regional liver function. Goshima *et al* (30) demonstrated that the liver-to-spleen volumetric ratio and contrast enhancement index on Gd-EOB-DTPA-enhanced MRI may be reliable biomarkers for determining the stage of hepatic fibrosis. Takao *et al* (31) reported that, following Gd-EOB-DTPA administration, the signal intensity in the bile duct may indicate underlying liver function. All of the abovementioned studies indicated that Gd-EOB-DTPA-enhanced MRI could be used to evaluate liver function, which is similar to the findings of the present study.

As proposed by previous studies, the current study used SNR to quantify the absolute and dynamic contrast enhancement due to its function as a surrogate marker of hepatocytic Gd-EOB-DTPA uptake (32). Quantification of functional impairment is reflected by the SNR value calculated for peritumoral regions and healthy liver tissue regions. The SNR values calculated for peritumoral regions and non-involved liver tissue showed no statistically significant difference on T1 unenhanced scans. However, the liver tissue in the peritumoral region exhibited significantly lower Gd-EOB-DTPA uptake compared with a similar region in healthy liver tissue 20 min after Gd-EOB-DTPA administration, indicating regional hepatocyte impairment caused by TACE therapy. It may be argued that the observed reduction in uptake in peritumoral regions may be due to the presence of tumor tissue. However, T2-weighted imaging of the same region revealed no significantly increased signal intensity, as would be exhibited by tumor tissue. Therefore, it is probable that this decreased signal intensity is due to ischemia following TACE therapy, and not the presence of tumor tissue.

Notably, relative SNR in the peritumoral region only correlated with the quantity of iodized oil used. The age, gender and Child-Pugh class of the patient, and the diameter and blood supply of the tumor did not correlate with relative SNR. This indicates that the dosage of iodized oil used in TACE therapy may determine the subsequent level of functional impairment of the liver, and must be considered when performing the procedure. The most likely cause of hepatocytic impairment is infarction of the hepatic artery by iodized oil, which leads to hypoperfusion of the peritumoral liver tissue (33). As the liver has a characteristic double blood supply, recovery from this impairment may be possible in the long term (34).

The present study had several limitations. Firstly, the number of patients included in this single center study was relatively small (n=29), which limited the power of the data analysis. Secondly, histological confirmation of regional hepatic damage was not included. The impairment of the liver tissue may have been caused by procedure-related cell death, parenchymal hypoperfusion and infarction of the liver tissue; histological examination is required to determine the actual cause of regional hepatic damage at a cellular level. Finally, as the patients may have had different underlying diseases, a bias was introduced by the potential varying disease mechanisms. In future studies, homogenous patient populations should be utilized.

In conclusion, the present data demonstrates an apparent decrease in Gd-EOB-DTPA uptake in peritumoral liver regions compared with healthy liver tissue regions 20 min after Gd-EOB-DTPA administration, indicating the existence of regional hepatocytic injury caused by TACE therapy. Gd-EOB-DTPA-enhanced MRI may therefore represent an effective, non-invasive tool for evaluating regional liver function impairment following TACE therapy. Furthermore, an association was observed between the relative SNR and the quantity of iodized oil used, indicating that the dosage of oil used may impact the subsequent level of functional liver impairment. Further study should be concerned with the timing of liver function recovery after TACE therapy using Gd-EOB-DTPA-enhanced MRI.

## Figures and Tables

**Table I:** Clinical data of patients for estimation.

	Age, years	Tumor diameter, cm	Quantity of iodized oil used, ml	Child-Pugh score
Mean	49.86	6.94	16.14	6.21
SD	11.05	2.15	7.04	0.94
Range	28–76	3.1–9.9	5–30	5–9

SD, standard deviation.

**Table II:** Signal to noise ratio in healthy and peritumoral liver tissue before and 20 min after Gd-EOB-DTPA administration.

	Before administration^a^			20 min after administration^b^		
		
	Healthy region	Peritumoral region		Healthy region	Peritumoral region	
Mean	50.53	49.81		82.55	75.77	
SD	15.99	15.85		33.33	27.41	
Range	11.25–83.46	12.34–81.53	P=0.191	31.45–153.02	31.42–144.49	P<0.001

a Before Gd-EOB-DTPA administration;

b 20 min after Gd-EOB-DTPA administration.

Gd-EOB-DTPA, gadolinium ethoxybenzyl diethylenetriamine pentaacetic acid.

**Table III:** Correlation between relative SNR in peritumoral regions and its influencing factors.

	Age	Gender	Tumor diameter	Blood supply	Child-Pugh class	Iodized oil dosage
Relative SNR	r=0.151	r=−0.381	r=0.226	r=0.241	r=0.106	r=0.528
	P=0.434	P=0.055	P=0.238	P=0.209	P=0.584	P=0.003^a^

a Relative SNR only correlated with the quantity of iodized oil used during transcatheter arterial chemoembolization therapy.

SNR, signal to noise ratio; Gd-EOB-DTPA, gadolinium ethoxybenzyl diethylenetriamine pentaacetic acid.

## Abbreviations

TACE: transcatheter arterial chemoembolization
HCC: hepatocellular carcinoma
Gd-EOB-DTPA: gadolinium ethoxybenzyl diethylenetriamine pentaacetic acid
MRI: magnetic resonance imaging
CT: computed tomography
ROI: region of interest
SI: signal intensity
SNR: signal to noise ratio
SD: standard deviation
ICG-PDR: plasma disappearance rate of indocyanine green
OATP: organic anion transporting polypeptide
